# Mosquito (Diptera: Culicidae) assemblages associated with *Nidularium *and *Vriesea *bromeliads in Serra do Mar, Atlantic Forest, Brazil

**DOI:** 10.1186/1756-3305-5-41

**Published:** 2012-02-16

**Authors:** Tatiani C Marques, Brian P Bourke, Gabriel Z Laporta, Maria Anice Mureb Sallum

**Affiliations:** 1 Departamento de Epidemiologia, Faculdade de Saúde Pública, Universidade de São Paulo, Av. Dr. Arnaldo 715, CEP 01246-904, São Paulo-SP, Brasil

**Keywords:** Culicidae, *Vriesea*, *Nidularium*, bromeliads, species co-occurrence, *Anopheles cruzii*, *Anopheles homunculus*, *Plasmodium *vectors, Atlantic Forest

## Abstract

**Background:**

The most substantial and best preserved area of Atlantic Forest is within the biogeographical sub-region of Serra do Mar. The topographic complexity of the region creates a diverse array of microclimates, which can affect species distribution and diversity inside the forest. Given that Atlantic Forest includes highly heterogeneous environments, a diverse and medically important Culicidae assemblage, and possible species co-occurrence, we evaluated mosquito assemblages from bromeliad phytotelmata in Serra do Mar (southeastern Brazil).

**Methods:**

Larvae and pupae were collected monthly from *Nidularium *and *Vriesea *bromeliads between July 2008 and June 2009. Collection sites were divided into landscape categories (lowland, hillslope and hilltop) based on elevation and slope. Correlations between bromeliad mosquito assemblage and environmental variables were assessed using multivariate redundancy analysis. Differences in species diversity between bromeliads within each category of elevation were explored using the Renyi diversity index. Univariate binary logistic regression analyses were used to assess species co-occurrence.

**Results:**

A total of 2,024 mosquitoes belonging to 22 species were collected. Landscape categories (pseudo-F value = 1.89, p = 0.04), bromeliad water volume (pseudo-F = 2.99, p = 0.03) and bromeliad fullness (Pseudo-F = 4.47, p < 0.01) influenced mosquito assemblage structure. Renyi diversity index show that lowland possesses the highest diversity indices. The presence of *An. homunculus *was associated with *Cx. ocellatus *and the presence of *An. cruzii *was associated with *Cx. neglectus, Cx. inimitabilis fuscatus *and *Cx. worontzowi. Anopheles cruzii *and *An. homunculus *were taken from the same bromeliad, however, the co-occurrence between those two species was not statistically significant.

**Conclusions:**

One of the main findings of our study was that differences in species among mosquito assemblages were influenced by landscape characteristics. The bromeliad factor that influenced mosquito abundance and assemblage structure was fullness. The findings of the current study raise important questions about the role of *An. homunculus *in the transmission of *Plasmodium *in Serra do Mar, southeastern Atlantic Forest.

## Background

The tropical forest of eastern South America, known as Atlantic Forest, is one of the world's most important biodiversity hotspots [1]. The most substantial and best preserved area of Atlantic Forest is found in the biogeographical sub-region of Serra do Mar [2], which contains approximately 30% of the remaining forest [3]. Although climatic conditions are relatively uniform across Serra do Mar, the topographic complexity of the region [4] creates a diverse array of microclimates. These topographical differences provide gradients of oxygen, humidity and temperature that can affect species distribution and diversity inside the forest [5,6]. As such, topography is likely to be an important factor in shaping floral [4] and faunal [6] diversity in Atlantic Forest.

A rich diversity of species from the Culicidae family is found in Serra do Mar [7,8]. Many of these mosquito species appear to have evolved in close association with bromeliads [9]. For example, *Culex ocellatus *Theobald and many species of *Culex *(*Microculex*) Theobald [10,11] and the subgenera *Hystatomyia *Dyar and *Phoniomyia *Theobald of the genus *Wyeomyia *are highly dependent upon bromeliads for larval habitat [9]. With the exception of *Anopheles *(*Kerteszia*) *bambusicolus *Komp, which is associated with bamboo internodes, the larva and the pupa of *Kerteszia *Theobald species depend on bromeliad phytotelmata from preserved environments as their primary larval habitat [12]. The larvae of *Anopheles *(*Kerteszia*) *cruzii *Dyar & Knab, *Anopheles *(*Kerteszia*) *bellator *Dyar & Knab, and *Anopheles *(*Kerteszia*) *homunculus *Komp are frequently found in *Nidularium *and *Vriesea *bromeliads in Atlantic Forest [13].

Human malaria is endemic in Serra do Mar [14] where the primary vectors are *An. cruzii *and *An. bellator *[15]. *Anopheles homunculus *has also been incriminated as a vector of human *Plasmodium *parasites in Paraná and Santa Catarina states [16]. Despite the medical importance of these three species, many aspects of their biology are poorly known [15]. Consequently, studying the way in which environmental variables influence the presence and distribution of these species may help to determine the role of vectors in the dynamics of human plasmodium transmission in Serra do Mar.

Considering the existence of environmental determinants for mosquito assemblages in larval habitats [17], and a highly heterogeneous environment [4] and diverse medically important Culicidae assemblage in Serra do Mar [7], the main objectives of the study are to: (1) characterize bromeliad mosquito assemblage structure; (2) assess correlations between bromeliad mosquito assemblage structure and various environmental factors; (3) determine correlations between the most abundant species and various bromeliad characteristics; and (4) assess co-occurrence among species.

## Methods

**Figure 1:**
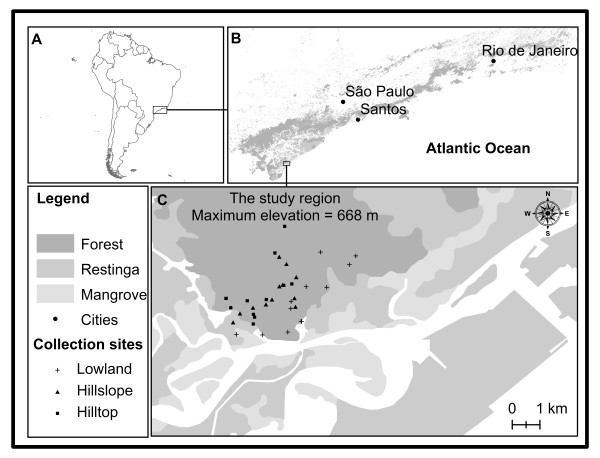
**Study area and collection sites. **A: Location of study area in South America. B: The remnants of Atlantic Forest in Serra do Mar, São Paulo and Rio de Janeiro states. C: The study area showing the collection sites according to landscape categories: lowland, hillslope and hilltop. The range of elevation (0 to 1,514 m) is relative to Serra do Mar (B).

The study area is located in the Aroeira District (25° 0' 54"S and 47° 55' 37"W, SAD 69) of Cananéia, Serra do Mar, São Paulo state (Figure 1). Collection sites were divided into three distinct landscape categories based on elevation, slope and site accessibility. They were: 1) lowland (5-20 m altitude), 2) hillslope (33 to 54 m altitude), and 3) hilltop (81 to 263 m altitude). All three sites were of primary and considerably preserved forest. The lowland area was found adjacent to an estuarine channel and mangrove. The area was very humid, with a high tree density and little light penetration at ground level. Rocky outcrops were scattered in this area, but abundant at higher elevations (hillslope and hilltop), which may have lowered humidity and increased light penetration at the hilltop.

Larvae and pupae were collected from terrestrial, epiphytic and saxicolous *Nidularium *(Subfamily: Bromelioideae) and *Vriesea *(Subfamily: Tillandsioideae) bromeliads in each of the three elevation categories at monthly intervals from July 2008 until June 2009. This yielded samples from 36 plants in each category, totaling 108 bromeliads. Monthly sampling sites varied spatially (by approximately 500 m) in order to increase the coverage area. Of the 108 bromeliads sampled, 94 were *Nidularium *and 14 *Vriesea*. Plants of both genera were equally sampled among elevation categories. Thirty *Nidularium *bromeliads were sampled on the lowland, 33 on the hillslope and 31 on the hilltop whilst 6 *Vriesea *were sampled on the lowland, 3 on the hillslope and 5 on the hilltop.

Data taken for each sampled plant were: type of bromeliad (terrestrial, epiphytic and saxicolous), bromeliad height, water pH, tank diameter, tank depth, and water volume. Water from each bromeliad was removed with a manual suction pump and measured for the water volume. Additional fresh water was poured into each plant and the removal was again repeated. Samples from each bromeliad were kept in separate plastic containers and the suction pump was washed with both 70% ethanol and fresh water to avoid cross contamination between plants. Climatic data from the two weeks preceding the collection date were obtained from the Centro Integrado de Informações Agrometeorológicas - CIIAGRO (http://www.ciiagro.sp.gov.br/) station in Cananéia.

Larvae and pupae were taken to the laboratory and raised to adulthood for species identification. Larval and pupal exuviae were mounted on microscopy slides with Canada balsam. Whenever possible, male genitalia were used for species identification. Morphological identification was based on Lane and Whitman [18], Lane [19], Correa and Ramalho [20], Cotrim and Galati [21] and Forattini [22]. Nine specimens that could not be identified using Lane's [19] keys were registered as *Cx*. (*Mcx*.) sp1 according to Marques' et al. [11] informal nomenclature. For the purpose of the present study, we identified larvae with evident subapical swelling as *Cx. daumasturus *[23], a species that was formerly synonymized with *Cx. imitator imitator *[19]. However, the validation of the species needs further investigation. Identification of *An. cruzii *and *An. homunculus *was based on characters of the fourth-instar larva, pupa and adults following the characteristics proposed by Forattini [22] and Sallum et al. [24]. Adults associated with either larval or pupal exuviae were deposited in Coleção Entomológica de Referência da Faculdade de Saúde Pública da Universidade de São Paulo (FSP-USP).

Statistical analyses were performed in the program R 2.12 (http://www.r-project.org/), using the packages BiodiversityR [25], MASS [26], and epicalc [27]. Species richness was extrapolated using the second order Jackknife [28] in order to assess the sampling effort. This estimator performs best with respect to accuracy in both even and uneven communities [29]. The multivariate redundancy analysis (RDA) [30] was used to assess correlations between bromeliad mosquito assemblage structure and various environmental variables. These variables included collection site landscape category (m), mean temperature (°C), total precipitation of rain two weeks prior to collection (mm), type of bromeliad (terrestrial, epiphytic and saxicolous), bromeliad height from the ground (m), tank diameter (cm), tank depth (cm), water pH, bromeliad water volume (ml), and an estimate of bromeliad tank fullness (ml/cm; water volume divided by tank height).

The Renyi diversity index [31] was used to explore differences in species diversity among bromeliads within each category of elevation (lowland, hillslope, and hilltop). This index provided four further diversity indices: Total richness, Shannon-Weiner index, Simpson-Yule index, and the Berger-Parker index. The differences between landscape categories for each of these indices were then tested for statistical significance using Kruskal-Wallis test (p < 0.05).

Correlations between species abundance and both bromeliad fullness and landscape category were assessed based on the results from the RDA analysis. Univariate Gaussian regression analyses were performed to determine whether the bromeliad fullness had either a positive or negative contribution to species abundance (n = 108, p < 0.05). Univariate binomial negative regression analyses were then carried out for landscape categories (n = 108). Relative species abundance (prevalence ratio) values for hillslope and hilltop were estimated using the lowland category as the baseline. A prevalence ratio of one indicates the given species is not associated with any landscape category, whereas values of greater than one and less than one indicate a positive and negative association, respectively (p < 0.05, CI 95%).

Univariate binary logistic regression analyses (n = 108) were used to assess species co-occurrence in bromeliads. Species abundance was transformed into dummy variables (absence = 0, presence = 1), and the analyses of these variables provided one of three possible results; an odds ratio value of one indicates species are associated randomly, whereas odds ratio values of greater than one and less than one indicate a positive and negative association, respectively (p < 0.05, CI 95%).

## Results

Two thousand and twenty four mosquitoes belonging to 22 species were collected (Table 1). *Culex ocellatus *(429; 21.20%), *Cx*. (*Mcx*.) *imitator retrosus *(388; 19.17%), *Cx*. (*Mcx*.) *neglectus *(350; 17.29%), *Cx*. (*Mcx*.) *imitator imitator *(213; 10.52%), *An. homunculus *(201; 9.93%), *Cx*. (*Mcx*.) *inimitabilis fuscatus *(126; 6.23%), *An. cruzii *(106; 5.24%), *Cx*. (*Mcx*.) *worontozowi *(80; 3.95%) and *Cx*. (*Mcx*.) *aphylactus *(55; 2.52%) were the most abundant species.

**Table 1:** Species of Culicidae found in *Nidularium* and *Vriesea* bromeliads.

Species	Landscape cateogories			Total
		
	Lowland	Hillslope	Hilltop	
*Anopheles *(*Kerteszia*) *cruzii *Dyar & Knab	43	24	39	106
*Anopheles *(*Kerteszia*) *homunculus *Komp	98	62	41	201
*Culex ocellatus *Theobald	101	114	214	429
*Culex *(*Microculex*) *sp*1	7	2	0	9
*Culex *(*Microculex*) *reducens *Lane & Whitman	0	1	5	6
*Culex *(*Microculex*) *worontzowi *Pessoa & Galvão	11	10	59	80
*Culex *(*Microculex*) *daumasturus *(Kum)	4	0	1	5
*Culex *(*Microculex*) *imitator imitator *Theobald	165	35	13	213
*Culex (Microculex) imitator retrosus Lane & Whitman*	81	159	148	388
*Culex *(*Microculex*) *aphylactus *Root	20	20	15	55
*Culex *(*Microculex*) *inimitabilis fuscatus *Lane & Whitman	29	53	44	126
*Culex *(*Microculex*) *microphyllus *Root	0	4	14	18
*Culex *(*Microculex*) *neglectus *Lutz	199	113	38	350
*Culex *(*Microculex*) *intermedius *Lane & Whitman	8	0	0	8
*Culex *(*Microculex*) *pleuristriatus *Theobald	2	0	0	2
*Runchomyia *(*Runchomyia*) *theobaldi *(Lane & Cerqueira)	0	1	0	1
*Wyeomyia *(*Phoniomyia*) *davisi *(Lane & Cerqueira)	2	0	0	2
*Wyeomyia *(*Phoniomyia*) *galvaoi *(Correa & Ramalho)	4	3	0	7
*Wyeomyia *(*Phoniomyia*) *incaudata *(Root)	2	0	0	2
*Wyeomyia *(*Phoniomyia*) *palmata *(Lane & Cerqueira)	2	0	0	2
*Wyeomyia *(*Phoniomyia*) *pilicauda *Root	0	0	1	1
*Wyeomyia *(*Phoniomyia*) *theobaldi *(Lane & Cerqueira)	8	5	0	13

**Total abundance**	786	606	632	2024

**Total species richness**	18	15	13	22

Species of Culicidae collected as larvae and pupae, in *Nidularium *and *Vriesea *bromeliads combined, from lowland, hillslope and hilltop, in an area of Atlantic forest in the municipality of Cananéia, southeastern São Paulo state, Brazil, from July 2008 to June 2009.

The extrapolated species richness using the second-order jackknife estimator was 35 species. Of the 22 species collected, two were found to be singletons and four doubletons. Results of the RDA showed that landscape categories, bromeliad water volume and bromeliad fullness had a significant influence on the bromeliad mosquito assemblage structure (pseudo-F value = 1.89, p = 0.04; pseudo-F value = 2.99, p = 0.03; and pseudo-F value = 4.47, p < 0.01, respectively; Table 2).

**Table 2:** Correlation between bromeliad mosquito assemblage and environmental variables.

Environmental variable	Type of variable	Redundancy analysis
Mean temperature	Numeric, from 16.9 to 27.5°C	Pseudo-F = 0.69, d.f. = 106 (p = 0.63)
Total precipitation	Numeric, from 0.9 to 155.3 mm	Pseudo-F = 0.13, d.f. = 106 (p = 0.99)
Bromeliad height	Numeric, from 0 to 260 cm	Pseudo-F = 0.61, d.f. = 106 (p = 0.68)
Type of bromeliad	Factor, 3 levels (ground, epiphytic, rocky)	Pseudo-F = 1.49, d.f. = 106 (p = 0.13)
Water pH	Numeric, from 3.9 to 7.5	Pseudo-F = 0.41, d.f. = 106 (p = 0.86)
Tank diameter	Numeric, from 10 to 100 cm	Pseudo-F = 0.50, d.f. = 106 (p = 0.75)
Tank depth	Numeric, from 10 to 140 cm	Pseudo-F = 0.98, d.f. = 106 (p = 0.41)
Landscape categories	Factor, 3 levels (lowland, hillslope, hillside)	Pseudo-F = 1.89, d.f. = 106 (p = 0.04)^a^
Bromeliad water volume	Numeric, from 7 to 750 ml	Pseudo-F = 2.99, d.f. = 106 (p = 0.03)^a^
Bromeliad fullness (water divided by depth)	Numeric, from 0.2 to 17.5 ml/cm	Pseudo-F = 4.47, d.f. = 106 (p < 0.01)^a^

Results of the multivariate Redundancy analysis (RDA) showing correlations between bromeliad mosquito assemblage structure and environmental variables.

a = Significant result under the null hypothesis: Pseudo-F = 0 (p < 0.05).

**Figure 2:**
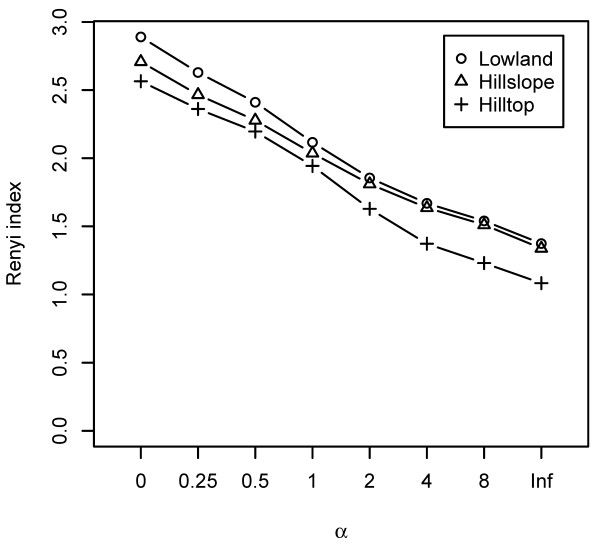
**Renyi index curves showing differences in species diversity between each bromeliad within each landscape category (lowland, hillslope, and hilltop).** The Renyi index estimates total richness for α = 0, Shannon-Weiner index for α = 1, the inverse Simpson-Yule index for ?α= 2 and 1/Berger-Parker index for α = Inf.

The Renyi diversity curves showed that the lowland possesses the highest diversity indices, and that the values for the hillslope are more similar to those obtained for the hilltop (Figure 2; Additional file 1). The Kruskal-Wallis tests indicated significant difference in the Total richness (= 0) and the Shannon-Weiner diversity index (= 1; Figure 2) between lowland and hilltop (KWχ^2 ^= 4.90, p = 0.03 and KWχ^2 ^= 5.75, p = 0.02, respectively). Further results are included in Additional file 1.

Univariate Gaussian linear regression analyses found that bromeliad fullness was positively associated with *Cx. imitator imitator *(*β*_1_= 0.90; p < 0.001), *An. cruzii *(*β *_1_= 0.19; p < 0.01), *Cx. neglectus *(*β *_1_= 0.68; p < 0.01) and *Cx. aphylactus *(*β *_1_= 0.10; p = 0.03). *Cx. inimitabilis fuscatus*, however, showed a negative association (*β *_1_= - 0.32; p = 0.04) indicating this species was more associated with shallow water inside bromeliad tanks (Additional file 2).

Univariate negative binomial regression analyses found that *An. homunculus *(prevalence ratio = 0.42; CI 95% = 0.18 - 0.95) and *Cx. neglectus *(prevalence ratio = 0.19; CI 95% = 0.07 - 0.5) were more closely associated with lowland than hilltop, and that *Cx. imitator imitator *was more closely associated with lowland than either hilltop (prevalence ratio = 0.08; CI 95% = 0.03 - 0.22) or hillslope (prevalence ratio = 0.21; CI 95% = 0.08 - 0.53) (Additional file 3).

Univariate binary logistic regression analyses for co-occurrence showed that *An. homunculus *was associated with *Cx. ocellatus *and *An. cruzii *was associated with *Cx. neglectus, Cx. inimitabilis fuscatus *and *Cx. worontzowi *(Table 3). Although *An. cruzii *and *An. homunculus *were occasionally found in the same bromeliad, this association was not statistically significant (1.82, p = 0.14). Considering mosquito frequency distribution among bromeliad genera (Additional file 4), results of co-occurrence analyses were mainly because of *Nidularium*. The number of plants sampled of the *Nidularium *genus was approximately seven times higher than those of the *Vriesea *(94 *Nidularium *versus 14 *Vriesea*).

**Table 3:** Co-occurrence between mosquito species in *Nidularium* and *Vriesea* bromeliads.

Species	**Odds ratio (95%CI)**, *Anopheles cruzii*	**Odds ratio (95%CI)**, *Anopheles homunculus*
*Anopheles homunculus*	1.82 (0.83, 4.01)	-
*Culex ocellatus*	0.84 (0.36, 1.95)	2.43 (1.06, 5.56)^a^
*Culex aphylactus*	1.68 (0.62, 4.58)	0.96 (0.36, 2.6)
*Culex imitator imitator*	1.22 (0.55, 2.73)	1.55 (0.71, 3.4)
*Culex imitator retrosus*	1.52 (0.69, 3.34)	1.23 (0.57, 2.64)
*Culex inimitabilis fuscatus*	3.73 (1.33, 10.5)^a^	2.1 (0.76, 5.83)
*Culex neglectus*	2.87 (1.28, 6.43)^a^	0.99 (0.46, 2.11)
*Culex worontzowi*	4.65 (1.33, 16.27)^a^	0.91 (0.29, 2.92)

Results of the univariate binary logistic regression analyses showing co-occurrence between mosquito species in *Nidularium *and *Vriesea *bromeliads combined.

a = Significant result under the null hypothesis: Odds Ratio = 1 (p < 0.05).

## Discussion and conclusions

The difference between the observed (n = 22) and extrapolated species richness (n = 35) in our study can be explained by the high number of rare species present in the tropical forest of Serra do Mar [4]. According to the second order jackknife estimator, we detected six rare species in our study, which constitutes approximately one quarter of the observed species. It therefore appears that rarer species were under-represented in our study, which may have affected our ability to detect the significance of some determinants (Table 2). However, those determinants that were significant for the assemblage structure were also significant for the most abundant species.

Alves et al. [4] found significant differences in forest structure and biomass variation along a 0-1100 m altitude gradient of coastal Atlantic Forest in Serra do Mar. The authors also showed how small changes in elevation in tropical regions can significantly affect various environmental factors such as air temperature, solar radiation, light availability, edaphic discontinuities, both soil moisture and temperature, nutrients availability, ground evaporation and microbial decomposition. Considering that environmental variables may have an impact on the availability and suitability of mosquito habitats, including larval habitats (e.g. bromeliads), one should expect to find differences in Culicidae assemblages in different locations within Atlantic Forest. In the present study, differences in mosquito community structure in a small gradient of altitude (0-263 m) were found, showing that the complexity of Serra do Mar and elevational gradient may be considered in ecological studies of Culicidae. This finding is consistent with that of Navarro et al. [32] who found that elevation is an important landscape determinant for Culicidae fauna distribution in Venezuela.

One of the main findings in our study was that differences in species among mosquito assemblages were influenced by elevation categories. The higher total species richness found in the lowland (Table 1) is consistent with greater niche availability, and may be related to higher species abundance because of the decreased probability of local population extinction [33]. Micro-climatic variation could not be evaluated because such data was not available but is likely to be important in influencing mosquito fauna and their distribution. The macro-climatic data that was used in our study could not explain differences in assemblage structure (Table 2).

Previous studies showed that differences in species among mosquito assemblages can be explained by bromeliad characteristics [34,35]. The quantity and quality of food resources, and physico-chemical properties of the water in bromeliads tanks may determine the species found in them [34]. It is noteworthy that pH, conductivity, temperature, and O_2 _concentration were found unrelated to both richness and species diversity of macro-invertebrate fauna inhabiting *Tillandsia turneri *Baker (Bromeliaces) in high altitude forest in Colombia [36]. However, bromeliad water volume and plant area were associated with abundance. Machado-Allison et al. [37] found a positive correlation between bromeliad structural complexity, habitat persistence, presence of predators and mosquito species richness. In another study, Araújo et al. [38] found that species abundance was positively associated with increased water volume, whereas richness was correlated with plant diameter. In considering that larval mosquito community structure may be influenced by both the volume of water inside a bromeliad tank and depth of the water, another variable named bromeliad fullness was assessed for *Vriesea *and *Nidularium *plants from Atlantic Forest. This variable was found to have a statistically significant influence on the mosquito community structure. Water fullness may create an array of micro-variation in the physico-chemical characteristics of water content, and thus in food resources and presence of predators that can affect mosquito larval community structure.

Mosquito species abundance may also be influenced by bromeliad taxa [9,39-42]. Navarro et al. [43] found mosquito taxa association with bromeliad family in Venezuela. Similarly, in Panaquire, Venezuela, Machado-Allison et al. [44] found that some species of mosquitoes were strongly associated with species of bromeliads. In the present study, among the six rarest mosquito species found in *Nidularium *and *Vriesea *bromeliads, four belonged to the *Wyeomyia *(*Phoniomyia*). Similarly, Müller and Marcondes [45] collected only a single individual of *Wyeomyia *(*Phoniomyia*) in plants of *Nidularium innocentii*. However, Mocellin et al. [46] found *Wyeomyia *(*Phoniomyia*) to be the most abundant taxa in *Neoregelia compacta *(Mez) and *Billbergia nana *E. Pereira in the Botanical Garden of Rio de Janeiro. It therefore appears that plants of the genus *Nidularium *do not represent important larval habitat for *Wyeomyia *(*Phoniomyia*). Given the large numbers of adult *Wyeomyia *(*Phoniomyia*) previously found in the study area [47], it is likely that this subgenus favors alternative bromeliad genera, for example, *Neoregelia *and *Billbergia*. Moreover, it is noteworthy that at least 21 additional genera of Bromeliaceae can be found in Serra do Mar [48], some of which may provide important larval habitat for *Wyeomyia *(*Phoniomyia*).

The subgenus *Kerteszia *is comprised of 12 species, of which four have been implicated as malaria vectors [12]. Three of these, *An. bellator, An. cruzii*, and *An. homunculus*, are important vectors in the Atlantic Forest. While *An. bellator *and *An. cruzii *are widely distributed, the geographical distribution of *An. homunculus *is poorly known [15]. Sallum et al. [24] stated that the lack of *An. homunculus *records in Brazil may be a consequence of an inability to effectively identify the species based on female morphological characters. Larval and pupal characteristics, on the other hand, are highly effective at resolving this species. Despite the abundance of *An. cruzii *and *An. bellator *found in the study area by Forattini et al. [49], we only identified *An. cruzii *and *An. homunculus*. The absence of *An. bellator *may be because of a preference for larval habitat either in the canopy [13] or restinga [50], but plants from both environments were not sampled. *Anopheles homunculus*, on the other hand, was frequently encountered in our study and made up approximately 65% of all *Kerteszia *specimens collected. *Anopheles homunculus *was similarly found to be common in enclosed, humid forests in Serra do Mar in Santa Catarina state [50]. It is noteworthy that Harbach and Navarro [51] reported *An. homunculus *in forests at altitudes of up to 1700 m in Auyantepui, Venezuela.

Great importance lies in the ability to effectively identify the presence of malaria vectors, and understand mechanisms involved in multiple species coexistence. The identification of a surrogate species may be used as a tool to assess the presence of vector species. In our study area, three species from the genus *Culex *were found to be indicative of the presence of *An. cruzii *and one was found to be indicative of *An. homunculus *(Table 3), and mostly within *Nidularium *plants (Additional file 4). However, the field observations conducted for the present study do not allow addressing ecological and evolutionary mechanisms of mosquito species coexistence. Consequently, hypotheses of species co-occurrence need further investigations employing an experimental design that combines field investigation and also mathematical modeling.

There are few published accounts of *An. homunculus *in the Atlantic Forest, and this study is the first to include this species in an assessment of diversity among mosquito assemblages. *Anopheles homunculus *seems to be abundant in *Nidularium *and *Vriesea *bromeliads, sharing the same plants with *An. cruzii*, and presence of both species were found to be associated with distinct categories of elevation and slope. The findings of the current study open questions about the importance of *An. homunculus *in the transmission of *Plasmodium *sp. in Atlantic Forest. The species was incriminated as a vector on coastal areas of Santa Catarina state [16], but its current role in human malaria transmission is largely unknown. It is noteworthy that *Alouatta *monkeys were found infected with *Plasmodium vivax, P. malariae *and *P. falciparum *in Serra do Mar, and thus they may act as reservoirs for human *Plasmodium *[52,53]. In considering that *An. homunculus *is found in areas with dense forest coverage [50] where *Alouatta *monkeys are present, it is plausible to propose that either the species, or other *Kerteszia *species, may be involved in the transmission of malaria parasites from monkeys to humans. The involvement of *An. homunculus *in the dynamics of malaria transmission in Atlantic Forest requires further investigation, with a particular emphasis on greater sampling and testing for infectivity across the region in different levels within the forest.

## Competing interests

The authors declare that they have no competing interests.

## Authors' contributions

TCM and MAMS conceived and designed the experiments. TCM did the field collections and identified the specimens. GZL performed the statistical analysis in the R package with contributions by BPB, MAMS and TCM. MAMS, TCM, BPB, GZL wrote the paper. All authors read and approved the final manuscript.

## Supplementary Material

Additional file 1 **Differences between landscape categories for each of α value of the Renyi index tested for statistical significance using Kruskal-Wallis test**. Results of Kruskal-Wallis test to assess statistical significance of Renyi index values. Click here for file

Additional file 2 **Results of univariate Gaussian regression analyses performed with species abundance against the bromeliad fullness (volume of water divided by depth of bromeliad tank)**. Results of univariate regression analysis to determine correlation between species abundance and bromeliad fulness defined as volume of water divided by depth of the bromeliad tank. Click here for file

Additional file 3 Univariate negative binomial models regression analyses of species abundance as a function of elevation categories with lowland as the baseline. Results of negative binomial regression analysis showing correlations between species abundance and elevation. Click here for file

Additional file 4 Species of Culicidae taken from the genera Nidularium and Vriesea. *Number of plants in which a species was found/number of plants sampled in a specific landscape category. List of species of Culicidae collected from Nidularium and Vriesea showing the number of plants in which a species was found and the number of plants sampled in a specific landscape category. Click here for file
