# Supplementary material for: Mosquito (Diptera: Culicidae) assemblages associated with Nidularium and Vriesea bromeliads in Serra do Mar, Atlantic Forest, Brazil
Source: Parasit Vectors. 2012 Feb 16;5:41. doi: 10.1186/1756-3305-5-41 (PMC3359275; doi:10.1186/1756-3305-5-41)
Supplement: Additional file 1 — Differences between landscape categories for each of α value of the Renyi index tested for statistical significance using Kruskal-Wallis test. Results of Kruskal-Wallis test to assess statistical significance of Renyi index values. [file 1756-3305-5-41-S1.DOC]

**Additional file 1. Differences between landscape categories for each of α value of the Renyi index tested for statistical significance using Kruskal-Wallis test.**

Results of Kruskal-Wallis test to assess statistical significance of Renyi index values.

| Renyi index (α) | lowland/hilltop | lowland/hillslope | hillslope/hilltop |
| --- | --- | --- | --- |
| 0 | KWχ^2^=4.90; p=0.03^a^ | KWχ^2^=3.36; p=0.07 | KWχ^2^=0.02; p=0.90 |
| 0.25 | KWχ^2^=5.27; p=0.02^a^ | KWχ^2^=3.28; p=0.07 | KWχ^2^=0.16; p=0.67 |
| 0.5 | KWχ^2^=5.27; p=0.02^a^ | KWχ^2^=3.45; p=0.06 | KWχ^2^=0.11; p=0.74 |
| 1 | KWχ^2^=5.75; p=0.02^a^ | KWχ^2^=3.54; p=0.06 | KWχ^2^=0.32; p=0.57 |
| 2 | KWχ^2^=4.13; p=0.04^a^ | KWχ^2^=2.32; p=0.13 | KWχ^2^=0.32; p=0.57 |
| 4 | KWχ^2^=3.60; p=0.06 | KWχ^2^=2.37; p=0.12 | KWχ^2^=0.24; p=0.63 |
| 8 | KWχ^2^=3.31; p=0.07 | KWχ^2^=1.92; p=0.17 | KWχ^2^=0.23; p=0.64 |
| Inf | KWχ^2^=3.26; p=0.07 | KWχ^2^=1.91; p=0.17 | KWχ^2^=0.25; p=0.62 |

^a^ = Significant result under the null hypothesis: KWχ2 = 0 (p < 0.05).
