# Supplementary material for: Mosquito (Diptera: Culicidae) assemblages associated with Nidularium and Vriesea bromeliads in Serra do Mar, Atlantic Forest, Brazil
Source: Parasit Vectors. 2012 Feb 16;5:41. doi: 10.1186/1756-3305-5-41 (PMC3359275; doi:10.1186/1756-3305-5-41)
Supplement: Additional file 2 — Results of univariate Gaussian regression analyses performed with species abundance against the bromeliad fullness (volume of water divided by depth of bromeliad tank). Results of univariate regression analysis to determine correlation between species abundance and bromeliad fulness defined as volume of water divided by depth of the bromeliad tank. [file 1756-3305-5-41-S2.DOC]

**Additional file 2. Results of univariate Gaussian regression analyses performed with species abundance against the bromeliad fullness (volume of water divided by depth of bromeliad tank).**

Results of univariate regression analysis to determine correlation between species abundance and bromeliad fulness defined as volume of water divided by depth of the bromeliad tank.

| Species | Regression coefficient and significance value of bromeliad fullness (ml / cm) |
| --- | --- |
| *Anopheles cruzii* | *β*_1_ = 0.19, p <0.01^a^ |
| *Anopheles homunculus* | *β*_1_ = 0.11, p = 0.36 |
| *Culex ocellatus* | *β*_1_ = 0.05, p = 0.89 |
| *Culex aphylactus* | *β*_1_ = 0.10, p = 0.03^a^ |
| *Culex imitator imitator* | *β*_1_ = 0.90, p < 0.001^a^ |
| *Culex imitator retrosus* | *β*_1_ = -0.36, p = 0.28 |
| *Culex inimitabilis fuscatus* | *β*_1_ = -0.32, p = 0.04^a^ |
| *Culex neglectus* | *β*_1_ = 0.68, p < 0.01^a^ |
| *Culex worontzowi* | *β*_1_ = 0.15, p = 0.31 |

^a^a = Significant result under the null hypothesis: *β*_1_= 0 (p < 0.05).
