# Supplementary material for: Mosquito (Diptera: Culicidae) assemblages associated with Nidularium and Vriesea bromeliads in Serra do Mar, Atlantic Forest, Brazil
Source: Parasit Vectors. 2012 Feb 16;5:41. doi: 10.1186/1756-3305-5-41 (PMC3359275; doi:10.1186/1756-3305-5-41)
Supplement: Additional file 3 — Univariate negative binomial models regression analyses of species abundance as a function of elevation categories with lowland as the baseline. Results of negative binomial regression analysis showing correlations between species abundance and elevation. [file 1756-3305-5-41-S3.DOC]

**Additional file 3. Univariate negative binomial models regression analyses of species abundance as a function of elevational categories with lowland as the baseline.**

Results of negative binomial regression analysis showing correlations between species abundance and elevation.

| Species | Prevalence ratio (95% CI),  hillslope / lowland | Prevalence ratio (95% CI),  hilltop / lowland |
| --- | --- | --- |
| *Anopheles cruzii* | 0.56 (0.22, 1.41) | 0.91 (0.37, 2.22) |
| *Anopheles homunculus* | 0.63 (0.28, 1.42) | 0.42 (0.18, 0.95) ^a^ |
| *Culex ocellatus* | 1.13 (0.28, 4.62) | 2.12 (0.52, 8.62) |
| *Culex aphylactus* | 1 (0.23, 4.4) | 0.75 (0.17, 3.35) |
| *Culex imitator imitator* | 0.21 (0.08, 0.53) ^a^ | 0.08 (0.03, 0.22) ^a^ |
| *Culex imitator retrosus* | 1.96 (0.65, 5.91) | 1.83 (0.61, 5.51) |
| *Culex inimitabilis fuscatus* | 1.83 (0.29, 11.64) | 1.52 (0.24, 9.7) |
| *Culex neglectus* | 0.57 (0.22, 1.43) | 0.19 (0.07, 0.5) ^a^ |
| *Culex worontzowi* | 0.91 (0.1, 7.94) | 5.36 (0.66, 43.46) |

^a^Significant result under the null hypothesis: Prevalence ratio = 1 (p < 0.05).
