# Supplementary material for: Mosquito (Diptera: Culicidae) assemblages associated with Nidularium and Vriesea bromeliads in Serra do Mar, Atlantic Forest, Brazil
Source: Parasit Vectors. 2012 Feb 16;5:41. doi: 10.1186/1756-3305-5-41 (PMC3359275; doi:10.1186/1756-3305-5-41)
Supplement: Additional file 4 — Species of Culicidae taken from the genera Nidularium and Vriesea. *Number of plants in which a species was found/number of plants sampled in a specific landscape category. List of species of Culicidae collected from Nidularium and Vriesea showing the number of plants in which a species was found and the number of plants sampled in a specific landscape category. [file 1756-3305-5-41-S4.DOC]

**Additional file 4. Species of Culicidae taken from the *Nidularium* and *Vriesea* bromeliads genera. *Number of plants in which a species was found/number of plants sampled in a specific landscape category.**

List of species of Culicidae collected from *Nidularium* and *Vriesea* bromeliads plants, showing the number of plants in which a species was found and the number of plants sampled in a specific landscape category.

| **Species/landscape** | **Lowland** | | | **Hillslope** | | | **Hilltop** | |
| --- | --- | --- | --- | --- | --- | --- | --- | --- |
|  | *Nidularium* | | *Vriesea* | *Nidularium* | *Vriesea* | | *Nidularium* | *Vriesea* |
| *Anopheles cruzii* | 12/30* | | 3/6 | 9/33 | 1/3 | | 15/31 | 0/5 |
| *Anopheles homunculus* | 19/30 | | 1/6 | 17/33 | 1/3 | | 13/31 | 1/5 |
| *Culex ocellatus* | 8/30 | | 1/6 | 10/33 | 3/3 | | 11/31 | 2/5 |
| *Culex* sp. 1 | 2/30 | | 2/6 | 1/33 | 0/3 | | 0/31 | 0/5 |
| *Culex* *reducens* | 0/30 | | 0/6 | 1/33 | 0/3 | | 2/31 | 0/5 |
| *Culex* *worontzowi* | 3/30 | | 1/6 | 5/33 | 0/3 | | 4/31 | 0/5 |
| *Culex* *daumasturus* | 1/30 | | 0/6 | 0/33 | 0/3 | | 1/31 | 0/5 |
| *Culex* *imitator imitator* | 21/30 | | 4/6 | 10/33 | 2/3 | | 3/31 | 0/5 |
| *Culex imitator retrosus* | 11/30 | | 1/6 | 17/33 | 1/3 | | 17/31 | 0/5 |
| *Culex aphylactus* | 7/30 | | 1/6 | 6/33 | 0/3 | | 5/31 | 0/5 |
| *Culex inimitabilis fuscatus* | 3/30 | | 0/6 | 7/33 | 0/3 | | 9/31 | 0/5 |
| *Culex microphyllus* | 0/30 | | 0/6 | 1/33 | 1/3 | | 4/31 | 0/5 |
| *Culex neglectus* | 20/30 | | 6/6 | 13/33 | 2/3 | | 9/31 | 0/5 |
| *Culex intermedius* | 0/30 | | 1/6 | 0/33 | 0/3 | | 0/31 | 0/5 |
| *Culex pleuristriatus* | 1/30 | | 0/6 | 0/33 | 0/3 | | 0/31 | 0/5 |
| *Runchomyia theobaldi* | 0/30 | | 0/6 | 1/33 | 0/3 | | 0/31 | 0/5 |
| *Wyeomyia* *davisi* | 0/30 | | 1/6 | 0/33 | 0/3 | | 0/31 | 0/5 |
| *Wyeomyia galvaoi* | 3/30 | | 0/6 | 3/33 | 0/3 | | 0/31 | 0/5 |
| *Wyeomyia incaudata* | 0/30 | | 1/6 | 0/33 | 0/3 | | 0/31 | 0/5 |
| *Wyeomyia palmata* | 1/30 | | 0/6 | 0/33 | 0/3 | | 0/31 | 0/5 |
| *Wyeomyia pilicauda* | 0/30 | | 0/6 | 0/33 | 0/3 | | 1/31 | 0/5 |
| *Wyeomyia theobaldi* | 3/30 | 3/6 | | 3/33 | | 1/3 | 0/31 | 0/5 |
